# Supplementary material for: Hydrodynamics and direction change of tumbling bacteria
Source: PLoS One. 2021 Jul 20;16(7):e0254551. doi: 10.1371/journal.pone.0254551 (PMC8291660; doi:10.1371/journal.pone.0254551)
Supplement: S1 File — The movies S1.avi, S2.avi and S3.avi are for the kinematic model of the tumble with θ0 = π/4, θ0 = π/3 and θ0 = π/2, respectively. (ZIP) [file pone.0254551.s001.zip › SM/supplementary_material.pdf]

Supplementary material for ‘Hydrodynamics and direction change of tumbling bacteria’

Mariia Dvoriashyna[1] and Eric Lauga[2]

Department of Applied Mathematics and Theoretical Physics, University of Cambridge, Cambridge, UK

1 Hydrodynamic resistance matrices

Here we give the values of the resistance matrices **A**, **B** and **D**, defined in Eqs. (16) and (18) of the main text. For convenience, we re-parametrise the helix from Eq. (2) in the main text as

$$\mathbf{r} = [R\cos(k\alpha s + \omega t), R\sin(k\alpha s + \omega t), \alpha s], \tag{1}$$

with $\alpha = P/l$ and $k = \pm 2\pi/l\alpha$ ($k > 0$ for a right-handed helix and $k < 0$ for a left-handed one). The velocity distribution along the filament is given by $\mathbf{u} = \mathbf{u}_d + \mathbf{U} + \boldsymbol{\Omega} \times \mathbf{r}$, where $\mathbf{u}_d$ is the deformation velocity given by

$$\mathbf{u}_d = \frac{\partial \mathbf{r}}{\partial t} = [-R\omega \sin(k\alpha s + \omega t), R\omega\cos(k\alpha s + \omega t), 0]. \tag{2}$$

Integrating the force distribution from Eq. (15) we obtain the results from Eqs. (16) and (18) with matrices whose non-zero entries are

$$A_{1,1} = L(-\zeta_\perp + \alpha^2 R^2/2(\zeta_\perp - \zeta_\parallel)k^2), \tag{3}$$
$$A_{2,2} = A_{1,1}, \tag{4}$$
$$A_{3,3} = L(-\zeta_\perp + \alpha^2(\zeta_\perp - \zeta_\parallel)), \tag{5}$$
$$B_{1,1} = \alpha^2 R^2(-\zeta_\perp + \zeta_\parallel)kL/2, \tag{6}$$
$$B_{1,2} = -\alpha L^2(\zeta_\perp/2 - \alpha^2 R^2(\zeta_\perp - \zeta_\parallel)k^2/4), \tag{7}$$
$$B_{2,1} = -B_{1,2}, \qquad B_{2,2} = B_{1,1}, \tag{8}$$
$$B_{3,3} = R^2\alpha^2(\zeta_\perp - \zeta_\parallel)kL, \tag{9}$$
$$D_{1,1} = L\left[2(\zeta_\perp - \zeta_\parallel)R^2\alpha^2(3 + k^2\alpha^2L^2) - 2\zeta_\perp(2\alpha^2L^2 + 3R^2)\right]/12, \tag{10}$$
$$D_{2,2} = D_{1,1}, \tag{11}$$
$$D_{3,3} = R^2L(-\alpha^2R^2k^2\zeta_\parallel + \zeta_\perp(-1 + \alpha^2R^2k^2)). \tag{12}$$

[1]Email: dvoriashyna@damtp.cam.ac.uk; ORCID: 0000-0002-6057-1919
[2]Email: e.lauga@damtp.cam.ac.uk; ORCID: 0000-0002-8916-2545

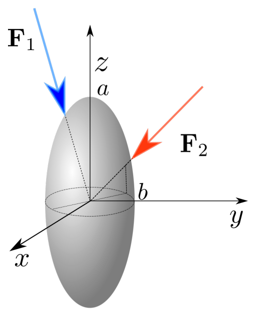

Figure 1: Spheroidal cell subject to two point forces $\mathbf{F}_1$ and $\mathbf{F}_2$ representing the propulsion from two 'phantom' flagella.

2 Forced-spheroid model

In this section we modify the model presented in § 4 of the main text to account for the spheroidal shape of the cell body. We consider the setup shown in Fig. 1. The cell body is modelled as a prolate spheroid with major axis a and minor axis $b < a$. The spheroid is pushed by N point forces $\mathbf{F}_1, \ldots, \mathbf{F}_N$, which represent 'phantom' filaments. The axes of the forces are chosen to pass through the centre of the spheroid, to eliminate possible force-generated torques, which, as we have shown in the discussion, are negligible.

As in the spherical case, we assume that the magnitude of these forces $|\mathbf{F}_i|$, $i = 1, \ldots, N$, coincide with propulsive forces of the helix with radius R_i and pitch angle β_i rotating with angular velocity ω_i [1],

$$|\mathbf{F}_i| = (\zeta_\perp - \zeta_\parallel) \sin\beta_i \cos\beta_i R_i L \omega_i. \tag{13}$$

In what follows we will consider magnitude of $\mathbf{F}_1$ to correspond to normal filament and $\mathbf{F}_2, \ldots, \mathbf{F}_N$ to semi-coiled ones. The total force acting on the cell body is $\mathbf{F}_t = \sum_{i=1}^{N} \mathbf{F}_i$. Moreover, $\mathbf{F}_1$ is assumed to coincide with the direction of the bundle. The velocity in the frame of cell body is now given by

$$\mathbf{U} = \mathbf{R}^{-1}\mathbf{F}_t, \tag{14}$$

with $\mathbf{R}$ being the resistance matrix for the spheroid, written in the (x, y, z) frame as

$$\mathbf{R} = \begin{pmatrix} C_x & 0 & 0 \\ 0 & C_y & 0 \\ 0 & 0 & C_z \end{pmatrix}, \tag{15}$$

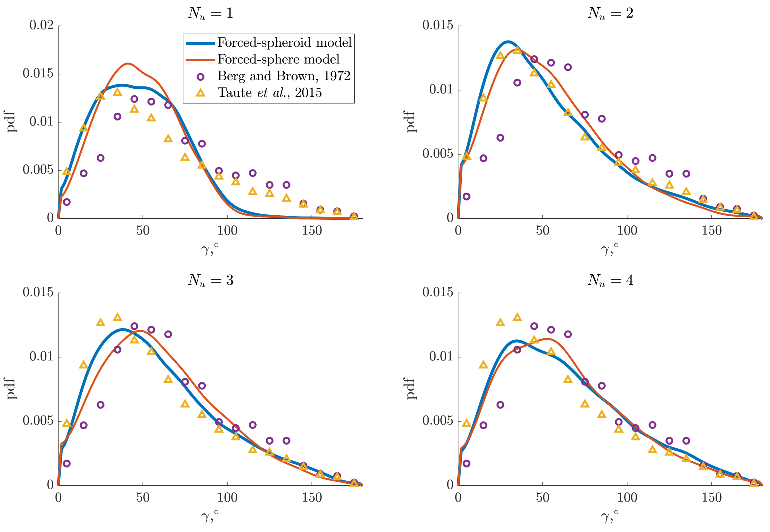

Figure 2: Probability density functions (pdfs) for the change-of-direction angle γ of a cell during a tumble for forced-sphere model (thin red solid line) and forced-spheroid model with aspect ratio $a/b = 2$ (thick blue solid line). In each cell, a number $N_u = 1$ to 4 forces come out of the bundle while the bundle remains aligned with $\mathbf{F}_1$. The thick blue line shows the results produced by the model described in this section, while the thin red line displays the results of the simplified forced-sphere model from § 4 in the main text. The experimental data of Berg and Brown [3] are shown in purple circles while the data of Taute *et al.* [4] are included in yellow triangles.

with

$$C_z = 16\pi\mu a e^3/(-2e + (1+e^2)E), \tag{16}$$

$$C_x = C_y = 32\pi\mu a e^3/(2e + (3e^2-1)E), \tag{17}$$

where $E = \ln\left(\frac{1+e}{1-e}\right)$ and $e = \sqrt{1-b^2/a^2}$ [2].

The total change of direction during a tumble, γ, is then given as the angle between $\mathbf{U}_0 = \mathbf{R}^{-1}\mathbf{F}_1$, which is swimming direction at $t < 0$ (i.e. before the tumble), and $\mathbf{U}$, i.e.

$$\gamma = \arccos\left(\frac{\mathbf{U}\cdot\mathbf{U}_0}{|\mathbf{U}||\mathbf{U}_0|}\right). \tag{18}$$

To obtain statistical predictions, we distribute forces uniformly on the surface of the cell using numerical rejection-sampling method for random points on a

spheroid [5]. We set $a = 2b = 1$ μm. Note that we assume that the axis of the 'previous' bundle coincides with $\mathbf{F}_1$, which is also chosen to be uniformly distributed on the cell body to avoid bias towards the poles of the cell. The probability density function obtained for γ with this model is shown in Fig. 2, with different panels corresponding to different number of forces, i.e. different number of semi-coiled filaments. As in the main text, the agreement with the experimental distribution is very good. Moreover, the red lines representing the results of the forced-sphere model are also in good agreement with those of the forced-spheroid model (maximum error in terms of mean values of γ is 3%), suggesting that including the spheroidal shape of the cell body does not drastically alter the results presented in the main text for a spherical cell.

References

[1] R. G. Cox. The motion of long slender bodies in a viscous fluid. Part 1. General theory. *J. Fluid Mech.*, 44:791–810, 1970.

[2] A. T. Chwang and T. Y. Wu. Hydromechanics of low-reynolds-number flow. part 2. singularity method for stokes flows. *J. Fluid Mech.*, 67:787–815, 1975.

[3] Howard C Berg and Douglas A Brown. Chemotaxis in escherichia coli analysed by three-dimensional tracking. *Nature*, 239:500–504, 1972.

[4] KM Taute, S Gude, SJ Tans, and TS Shimizu. High-throughput 3d tracking of bacteria on a standard phase contrast microscope. *Nat. Comm.*, 6:1–9, 2015.

[5] T. Chen and S. C. Glotzer. Simulation studies of a phenomenological model for elongated virus capsid formation. *Phys. Rev. E*, 75:051504, 2007.
