# Supplementary material for: Hydrodynamics and direction change of tumbling bacteria
Source: PLoS One. 2021 Jul 20;16(7):e0254551. doi: 10.1371/journal.pone.0254551 (PMC8291660; doi:10.1371/journal.pone.0254551)
Supplement: Attachment — Submitted filename: PloS ONE - answers to 1st round reviews.pdf [file pone.0254551.s002.pdf]

UNIVERSITY OF CAMBRIDGE

14 June 2021,

Dear Editor,

We are hereby submitting a revised version of our paper “Hydrodynamics and direction change of tumbling bacteria” to *PLoS ONE.*

We reply below to each of the referee’s comments and indicate where we have modified the paper in response; all changes to the paper appear in blue in the revised version. We have also added section 2 to the supplementary material.

We hope you will find the changes to our manuscript adequate and will be able to accept it for publication in *PLoS ONE*.

Best regards

Mariia Dvoriashyna
Eric Lauga

Replies to First Referee

This is a great piece of work that models the bundling and unbundling process of E.coli. Of course, I also find that there are so many assumptions adopted, which might affect the readability of the work.

We thank the referee for their positive appreciation of our work.

Here are a few minor points:

1. hight-handed -> right-handed, line 144.

Thank you, this has been corrected.

2. what is the difference between r in eq 1 and bar{r} in eq 2?

Thank you for this comment. In Eq 1, r is a position of the helix in the local coordinate system (\xi_1,\xi_2,\xi_3), while \bar{r} is in the frame of cell body, i.e. in the (x,y,z) coordinates. We have clarified this in the revised text.

3. line 162, i can understand that sliding can be enabled by elastic hooks, however, when this happens, it will be difficult to think that the filaments are still normal to the cell body? any supporting information for this?

We thank the referee for this comment, this is a good point. In reality the filaments do not always remain normal to the cell body, and their direction is governed by the elasticity of the hook, as described, e.g. in *Riley et al. (2018) Sci. Rep.,* ***8****, 10728*. In our model we strive for simplicity at every step so we assume that the filaments remain normal to the cell body, but the filaments are allowed to slide along the cell to simulate their different directions. This assumption of course preserves the forces that are applied on the body by the filaments but it does change the torques that are applied: indeed, every time a filament is not directed along the normal, it exerts a small torque on the centre of the cell body. However, as we explain in the discussion, the additional torques generated by the off-axis orientation of the filaments are negligible in terms of the overall reorientation angle, since their magnitude is much smaller than that required to reorient the cell and the filaments (these are cases (i) and (ii) in figure 10). We have added a comment in the revised paper to ensure that this assumption is clear.

4. in fig. 4, the definition of \psi can be difficult to understand, according to its definition on line 190

We thank the referee for noticing this. We have now added the beta_1 and beta_2 angles in the figure and the reference to the figure after the definition of \psi; hopefully this makes it clearer.

5. eq 6, could the authors provide more details on how this is derived? to me, this may not look that intuitive.

Thank you, we have added the details of the calculation in the text and modified further figure 4 to include the triangle that we used to calculate L_2.

Replies to Second Referee

In general, this paper is interesting. But there are some issues to be addressed.

We thank the referee for their positive appreciation of our work.

1. The authors intended to model E.coli which is a rod-shape bacteria. Why the authors use a spherical particle to represent the rod-shape bacteria? In recent years, the following paper has carefully addressed the modelling of Rod-shape bacteria in fluid flow. Modelling bacterial twitching in fluid flows: a CFD-DEM approach, Scientific Reports, 2019, 648915

We thank the reviewer for this comment. We now cite a number of numerical studies (including the one suggested by the reviewer) in the discussion section, where we propose possible extensions for different cell body shapes.

Regarding the choice of spherical shape, this was mainly done for the simplicity of the model (we have tried to make the simplest choice, if justified, at every stage of this modelling work). It can be shown, however, that the results of the spherical model are in good agreement with the results for a spheroid. Specifically, consider the case of the forced spheroid, as shown in the figure on the left with F_1, F_2 representing 'phantom' forces produced by normal and semi-coiled filaments (the spherical case is in the original paper). The axes of the forces are chosen to pass through the center of the spheroid, to eliminate possible force-generated torques, which, as we have shown in the discussion, are negligible in the estimate of reorientation angle (case (ii) in figure 10). The velocity in the frame of cell body is now given by $U = R^{-1}(F_1 + F_2)$ with R being the resistance matrix for the ellipsoid. The change of direction can be written as $\gamma = \arccos[(U, U_0)/|U||U_0|]$, where $U_0 = R^{-1}F_1$ is the swimming direction before the tumble. We distribute forces uniformly on the surface of the spheroid, and obtain the reorientation angles numerically.

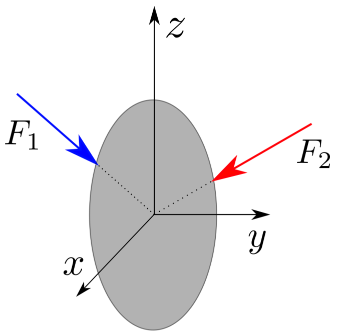

In the figure below we show pdfs of γ for a forced-spheroid model with aspect ratio 1:2 and forced-sphere model described in the manuscript. The agreement between the models is very good and the means differ only by several degrees. Therefore, the choice of spherical cell body is well justified.

We have included these results and the model description in the supplementary material with references to it in the main text.

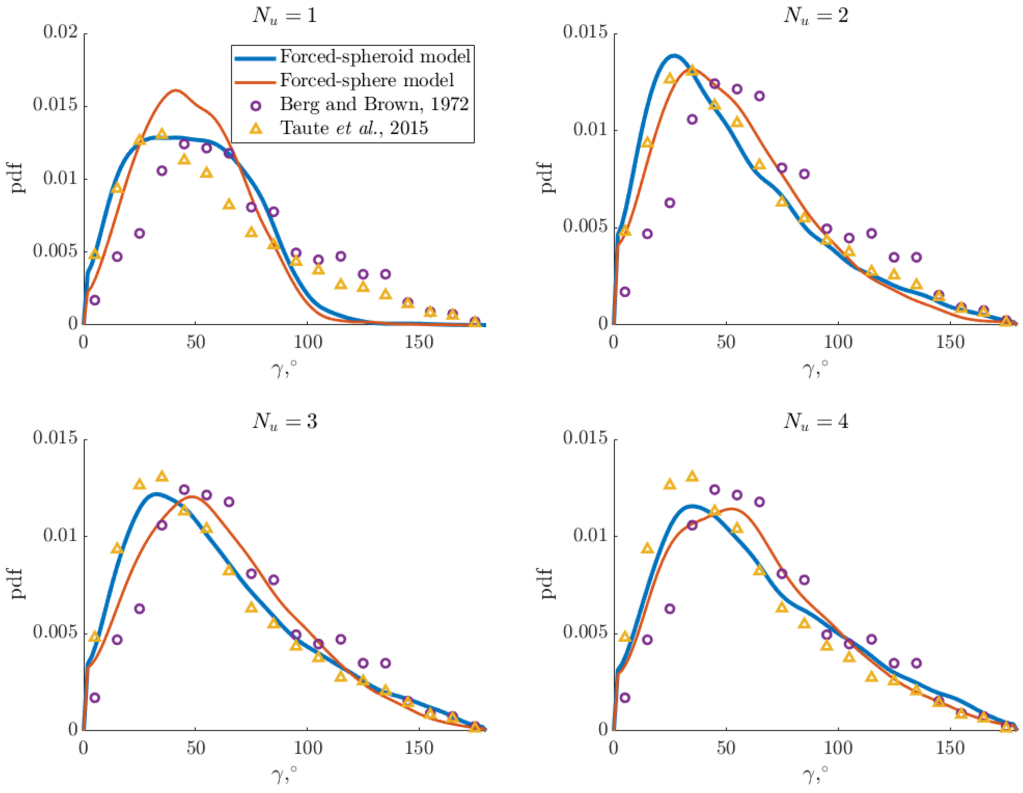

2. The manuscript is wordy and seems like a chapter of PhD thesis. It should be condensed.

We have tried to carefully go through the paper and make the revised version less wordy. Unfortunately, by its nature the paper is a very long construction of a model with many assumptions stated and discussed before the final results are obtained, so we feel like some of the wordiness of it is somewhat inevitable. Hopefully the new version is better though...

3. It would be better to clarify the new physical insights we could learn from the modelling, which cannot be obtained by experiments alone.

The paper demonstrates essentially one thing: the process of change of direction that is undertaken by swimming bacteria is fully governed by geometry and mechanics of the interacting flagella. In the paper, we use a geometrical description of the cells taken directly from experimental observations and use fluid mechanics to show that the change of direction can be fully predicted by what is happening at the flagellar physics level. We have tried in the revision to emphasise what we believe is an important physical insight.

Replies to Third Referee

General

The main shortcoming of the methodology is that there are a lot of ad hoc assumptions about the behaviour of the flagella in place of a mechanistic model. This is understandable for the sake of obtaining a simple conceptual model but raises questions about consistency and physicality.

We thank the referee for their careful reading of our paper. The purpose of our paper was to show that, using fluid mechanics and physics, we can make the direct link between the geometry of the interacting flagella and the change of direction undertaken by swimming bacteria. To do so, it does indeed take us a long time to construct a model and we have to make many assumptions since all parameters of the problem need to be accounted for.

For example, is it valid to allow the point of connection between the cell body and flagellum to slide around the cell? There should be a section of the flagellum that wraps around the body from the motor to the bundle and this should contribute to the force and torque balance equations. (Please see below for related remarks.)

Yes this is a good point, and it is true that this small section of the flagellum does not appear in our model. However, the small portion of the flagellar filament that is wrapped around the cell body is contributing to only a negligible amount of drag and propulsion, because of the no-slip boundary condition on the cell body, and hence its impact in the overall force and torque balance can be neglected. We have modified the paper to include the point raised by the referee and our response to it.

Furthermore, as we point out in the discussion, the torque needed to reorient the cell by a magnitude similar to that in the experiments is about an order of magnitude larger than that provided by the rotations of the filaments. Thus, even if the additional force/torque due to this extra portion of the filament was included, it would be too small to impact the angle γ.

Also, it appears from the model that the changing orientation of the bundle is largely responsible for the reorientation. Is there experimental evidence that the orientation of the bundle relative to the cell changes as in the model? If so, it would be surprising if the non-spherical shape of E. coli cells were not important. Presumably, there are preferred orientations, altering the distribution of random angle changes.

The referee raises a very valid point here. Unfortunately, it is impossible for us to extract statistically significant information from the available data to properly justify our modelling assumption that the bundle and the filament are coming together at a given axis. Instead, we based this assumption on several empirical events shown in the supplementary videos from the paper Turner *et al, J. Bacteriol* (2000)

(http://www.rowland.harvard.edu/labs/bacteria/movies/showmovie.php?mov=fluo_fil_leave), as we describe below using screenshots from the videos. We argue that these observations along with *a posteriori* agreement with experimentally measured turning angles justify this assumption.

For example, in the first bundling event of this video, we see the filaments and the bundle come together to join on the side of the cell body (frames 16-18):

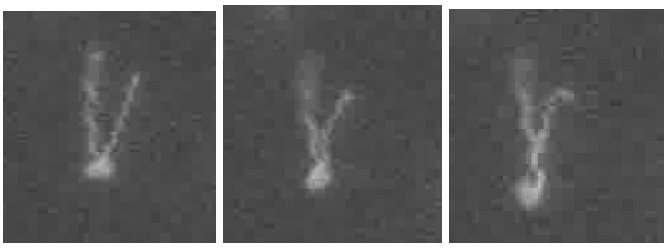

During this event the flexibility of both filament and the hook seem to play an important role, and we combine these two effects in our simple bundling model by allowing filament and bundle to join at a certain axis. For further observation, we look at the second bundling event from the movie (frames 42,44,48-52):

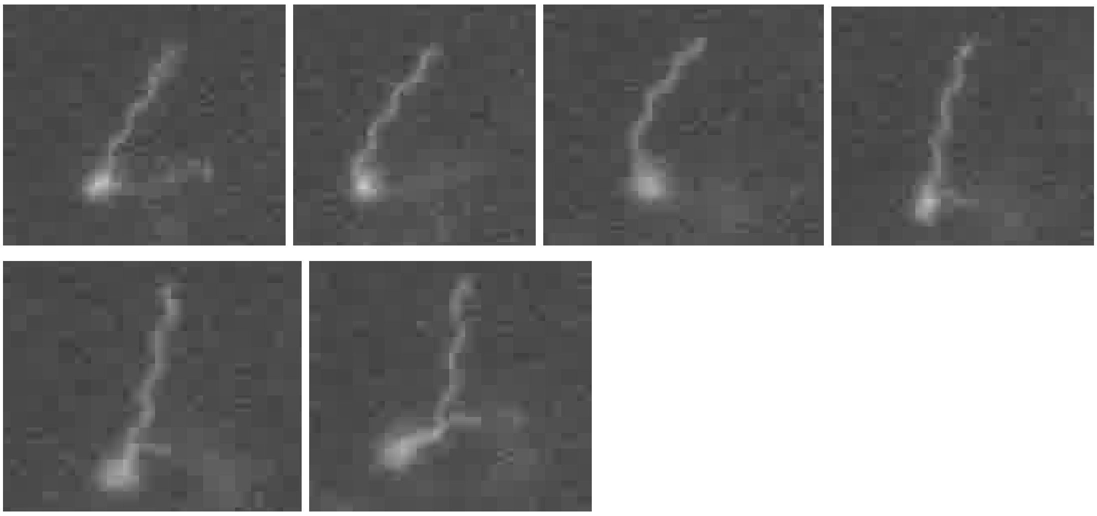

as well as the third bundling event (frames 124,126-128):

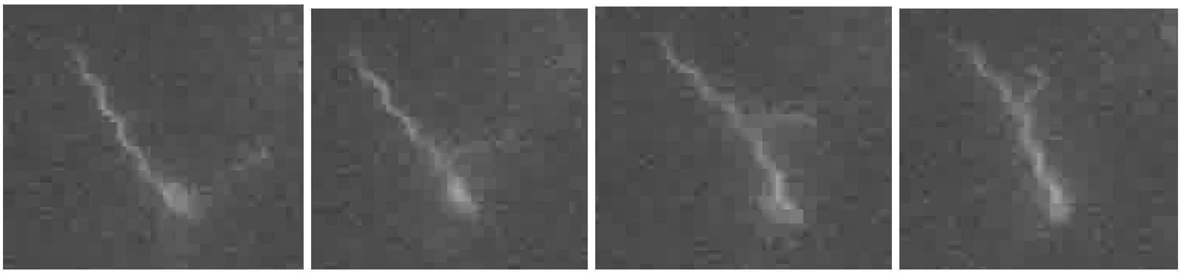

and are able to confirm similar behaviour of the filament and the bundle. We have added comments in the revised manuscript to make this clear.

Specific

1. The comparison with experiments focused on distributions of reorientation angle. Does the trajectory as a whole match experimental observations, e.g., during the semi-coiled propulsion phase?

We thank the referee for this point as well. It is unfortunately difficult to find the experimental information on the cell dynamics *during* tumble as it is occurring in such a short period of time, and since it is difficult to visualise the filaments. To properly compare trajectories we need to know the position of the filaments on the cell body and the tumble duration. Luckily, that is available from one of the videos mentioned above: event 2 is long enough to allow for a comparison, so we report below model results for the cell trajectory (axis are in meters $\cdot\ 10^{-7}$) while it is in semi-coiled propulsion (blue line), the position of the cell centre from the images (red symbols) and their linear fit (dashed red line).

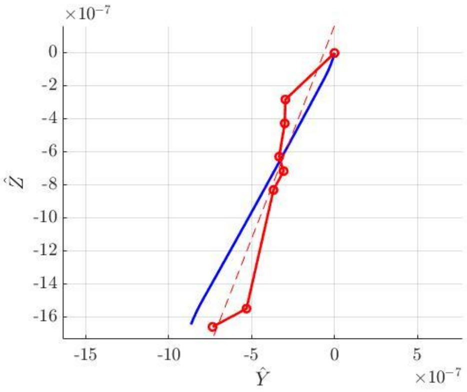

The quantitative agreement between the lines is satisfactory though of course there are many factors that the model is missing, including cell wobble. But unfortunately with the data we have this is the best we can do.

2. The figures depicting the model flagella are slightly misleading. The model considers pure helical segments, which do not connect to the cell body or to each other as a smooth curve. It should be noted that the representations in the figures are not the actual geometrical model of the filament.

Thank you, yes this is an important point, we did not intend to be misleading. In the revised paper we have changed the figure caption and the text to indicate that these are sketches but, as the referee correctly points out, the mathematical calculations only concern pure helical segments.

3. It is not clear why the kink velocity should be related to the rotation rate as in Eq. 7. It should be more complicated than this, e.g., dependent on the energy density of the two helical forms.

We acknowledge (and we now mention it in the revised text) that this does not take into account the elastic energy density of helices. Such helical transformations are complicated physical problems, which were thoroughly addressed by several solid-mechanics works (see e.g. Goldstein *et al*, *Phys. Rev. Lett.,* 2000 or Vogel and Stark, *Phys. Rev. Lett,* 2013). Insead we consider in this paper a simplified and analytically-tractable approach where we model the kink velocity based on purely geometrical arguments; our approach follows the classical work of Macnab and Orston, *J. Mol. Biol.* (1977).

4. In line 232 and a few other locations, there are remarks like "T_u is not necessarily -T_m". If the torque should be -T_m, why not choose omega_1 and omega_2 so that this is true?

We thank the reviewer for this comment. We should have been more precise in the original paper, and have now added several clarifications on the matter. In the equations 7 and 8 there are 3 unknowns: v_0, omega_1 and omega_2. As there are only 2 equations for them, one has to use one additional piece of information in order to fully determine the three values. We choose in the paper to fix the time of the (un)bundling, as it is an experimentally observable variable, which in turn fixes v0. This then results in small mismatches in torque, which we fix in an ad-hoc way by linearly connecting it to the known value of the motor torque. Alternatively, we could have prescribed the value of omega_2 so that it reaches T_m at the end of the (un)bundling process. This however, might provide (un)bundling times different from the experiments. The choice we made was dictated by our desire, throughout the paper, to agree with experimentally-observable variables. We have now stressed it in the revised manuscript.

Is this essentially what it means when "the torque changes linearly from T_u to -T_m"? Or is Eq. 3 being modified over time? Even though it is checked that the outcome is not sensitive to the choice of delta, I struggle to see why this step is necessary.

The result T=T_m in eq. 3 governs the angular velocities of the bundle and of the semicolied filament during normal swimming, but not during polymorphic transformation (this has now been specified in the paper). Therefore a linear change from T_u to -T_m is not governed by this equation; instead this is applied to enforce continuity of the torque after the filament has unbundled. It is not strictly necessary and one could have a jump in the torque with no real impact on the final results (we have checked this numerically), but it seemed to us to be more physical to have a smooth connection.

5. Is it physically justified to compute the torque on the flagella using Eq. 3 and setting $T_m = T$? The motor torque should be applied in a fixed direction (e.g., normal to the cell body), whereas the axes of the flagella are changing throughout the unbundling/bundling process and, moreover, the bundle moves so the total motor torque is different before and after tumbling. Is this possible if the motors are fixed on the cell body?

Setting the torque as $T=T_m$ is straightforward to implement when the filaments are not transforming, i.e. when they are in the bundle and in the semi-coiled stage and each filament is both straight and aligned with the axis of the motor. In any other situation, the direction of applied torque is determined by the hook, which connects the motor to the filament, and one would have to carefully think about force and moment balance in all situations where the helical axis is not aligned with the direction of the motor. In our model, during (un)bundling of course a torque continues to be applied but we did not fix the torque condition (dynamics) but instead we prescribed the kinematics of the transformation (geometry), as mentioned in the replies above.

6. In Eq. 22, should the omega at the start of the LHS be omega_i?

Thank you, this has now been corrected in the revised version.

The segment lengths l_i change with time but my interpretation is that time averaging is done for a fixed l_i at each instant in time. Is this correct?

We thank the reviewer for spotting this error, which we have now corrected by swapping the time averaging and space integral in the revised manuscript.
